# Reliability of photographic analysis of wound epithelialization assessed in human skin graft donor sites and epidermolysis bullosa wounds

**DOI:** 10.1186/s13063-015-0742-x

**Published:** 2015-05-28

**Authors:** Hans-Oliver Rennekampff, Rolf Fimmers, Hans-Robert Metelmann, Hauke Schumann, Mayer Tenenhaus

**Affiliations:** Department of Plastic Surgery, Hand Surgery, Burn Center, University Hospital, RWTH University Aachen, Aachen, Germany; Institute of Medical Biometry, Informatics and Epidemiology (IMBIE), University of Bonn, Bonn, Germany; Department of Oral and Maxillofacial Surgery/Plastic Surgery, Medical School, Ernst Moritz Arndt University, Greifswald, Germany; Medical Education, Catholic University of Applied Sciences Freiburg, Freiburg, Germany; Department of Plastic and Reconstructive Surgery, UC San Diego Health System, 4510 Executive Drive, San Diego, CA 92121 USA

**Keywords:** Clinical study, Epithelialization, Observer blind, Reliability, Telemedicine, Wound, Wound photography

## Abstract

**Background:**

In many clinical trials on cutaneous healing, wound closure is the primary endpoint and single most important outcome parameter, making precise assessment of this time point one of utmost importance. The assessment of wound closure can be performed either by subjective clinical inspection or with a variety of methodologies anticipated to provide more objective data. The aim of this study was to examine intra- and interrater variability of blinded photographic analysis of wound closure of human partial thickness wounds, as well as the reliability of remote photographic analysis of wounds with that of direct clinical assessment.

**Methods:**

Two plastic surgeons, a dermatologist, and a maxillofacial surgeon constituted our rater panel. High-resolution images of patient wounds derived from two randomized controlled clinical trials (EU Clinical Trials Register numbers EudraCT 2009-017418-56 (registered 12 January 2010) and EudraCT 2010-019945-24 (registered 13 July 2010)) were individually assessed by the blinded, experienced study raters. The reliability of photographic image analysis was tested using intraclass and interclass correlation. The validity of photographic image analysis was correlated with clinical assessments of documented time to heal from the study centers’ files.

**Results:**

The results demonstrated that the mean intraclass correlation coefficient of all four examiners was excellent (*r* = 0.79; 95 % confidence interval (CI), 0.61, 1.00)). The interrater correlation coefficient was good (*r* = 0.67; 95 % CI, 0.57, 1.00)) and therefore acceptable. The agreement between remote visual assessment and clinical assessment at the time of healing was good (*r* = 0.64; 95 % CI, 0.52, 0.76)) with an overall difference of about 1 day.

**Conclusions:**

Remote photographic analysis of cutaneous wounds is a feasible instrument in clinical open-label studies to evaluate time to wound closure. We found that it was a reliable method of measuring wound closure that correlated satisfactorily with clinical judgment, bolstering the potential relevance in the current era of evolving application and dependency in the field of telemedicine.

**Trial registration:**

EU Clinical Trials Register EudraCT numbers 2009-017418-56 (date of registration: 12 January 2010) and 2010-019945-24 (date of registration: 13 July 2010).

## Background

Complete reepithelialization of cutaneous wounds is one of the most important time points in clinical wound healing. In many clinical trials on cutaneous healing, wound closure is the primary endpoint and single most important outcome parameter, making precise assessment of this time point one of utmost importance. The assessment of wound closure can be performed either by subjective clinical inspection or with a variety of methodologies anticipated to provide more objective data.

Subjective assessment is based upon the gross clinical appearance of the wound by experienced observers [1]. Traditionally, one characterizes the newly epithelialized wound as presenting with a dry (non-desiccated), glistening, silvery surface. A variety of invasive and non-invasive methods have been described for objective measurement of reepithelialization. Skin biopsies are considered one such objective standard; yet, the determination of which site to biopsy remains subjective to the examiner [1]. A similar challenge results when evaluating other promoted objective measurements of epithelialization such as electrical impedance [2] and transepidermal water loss [3], among others. Even with these quantitative modalities, identifying and choosing a region of interest may not necessarily represent the whole of the wound area. This inherent imprecision is particularly confounding in open-label randomized clinical studies, where the ultimate choice of the region of interest might well bias the study outcome. Furthermore, blinding of the evaluating clinicians at the study site is often intricate or even impossible.

In an effort to exclude bias of the region of interest, digital image analysis [4], optical coherence tomography [5] and light image technologies have all been advocated as more methods of objectively evaluating and assessing the complete wound area. To be clinically useful for study, these assessment techniques must be both reliable and valid [4, 6]. *Reliability* refers to the outcome‘s precision or repeatability on repeated testing. Interrater reliability assesses the consistency of the data when the same parameter is measured by different investigators using the same tool. Intrarater reliability describes the difference between the assessed parameter by a single observer when the parameter has not changed. Intra- and interrater reliability are determined by performing intraclass correlation coefficient analysis and should exceed a value of 0.75 to establish excellent reliability. Values between 0.4 and 0.75 represent fair to good reliability [7]. *Validity* refers to the ability of the outcome parameter to measure what is intended to measure, such as clinical epithelialization.

The present study was intended to determine the intra- and interrater reliability of four independent and experienced observers in assessing photographic records of wound sites. In our study, validity should determine the correlation between photographic records and the reported clinical assessment of the wound.

## Methods

### Study design

We performed a secondary analysis of data obtained from two prospective, open-label randomized controlled clinical trials in which researchers assessed the effect of a triterpene extract from birch cork on wound healing. Both studies were approved by local institutional review boards (see below). The first study was performed on split-thickness skin graft donor sites, with each donor site divided into halves and one half treated with a non-adhesive foam dressing (Mepilex; Mölnlycke Health Care, Gothenburg, Sweden) alone and the other half treated with Oleogel-S10 ointment (Birken AG, Niefern-Öschelbronn, Germany) covered by Mepilex (EudraCT number: 2009-017418-56; ClinicalTrials.gov identifier: NCT01294254; ethics committee of the Ernst Moritz Arndt University, Greifswald, Germany; Ethics Committee of the Albert Ludwigs University, Freiburg, Germany). The second study, also with an intraindividual comparison, included wounds of patients with epidermolysis bullosa. The wounds in these patients were also treated in matched pairs (either two halves of one wound or two comparable wounds simultaneously in the same patient) with verum or a control non-adhesive wound dressing (EudraCT number: 2010-019945-24; ClinicalTrials.gov identifier: NCT01294241; ethics committee of the Albert Ludwigs University, Freiburg, Germany).

Informed consent was obtained from all participating patients. The primary study protocol of both studies included high-quality, high-resolution photographic images at each dressing change until day 14. The degree of epithelialization assessed by the treating physician was recorded as part of the primary study protocol. The original photographs were macro photographs showing the wound and the surrounding skin peripherally labeled with information documenting patient number, wound orientation and study treatment regimens.

**Fig. 1:**
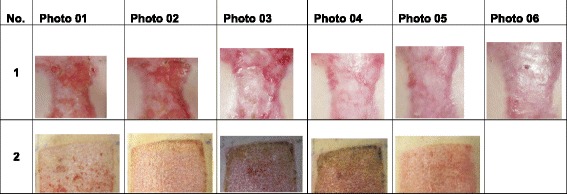
Two representative series of photographs series of forty-seven series evaluated. Four expert reviewers were independently asked to answer, for each series, which photograph was the first one to show a closed wound (minimally 95 % epithelialized)

To eliminate any potential bias, pictures were cropped to expose only wound areas of a single treatment regime and no skin markings were shown. All available pictures of a wound area were arranged in chronological order. Pictures were labeled with consecutive numbers, and no information was provided on the date or the time elapsed since first treatment of the wound (Fig. 1).

### Measures and outcomes

Photographic records of the 2 clinical studies, which included a total of 47 different wound halves, were evaluated by 4 experienced clinicians each with at least 15 years of experience in treating cutaneous wounds of different origin. The clinicians were two plastic surgeons, one dermatologist and one maxillofacial surgeon. The images, which were digitally sent to the four evaluators, had a resolution of approximately 100 × 160 pixels.

The examiners were asked to identify the image within a series (Fig. 1) where wound closure was judged to be complete. A wound exhibiting greater than or equal to 95 % of the surface as epithelialized was deemed closed. Cases where incomplete reepithelialization occurred were recorded as such. Three weeks after the first assessment, the same images were reassessed by all four participants according to the same procedure. The time interval of 21 days was chosen to avoid memory bias [8].

**Fig. 2:**
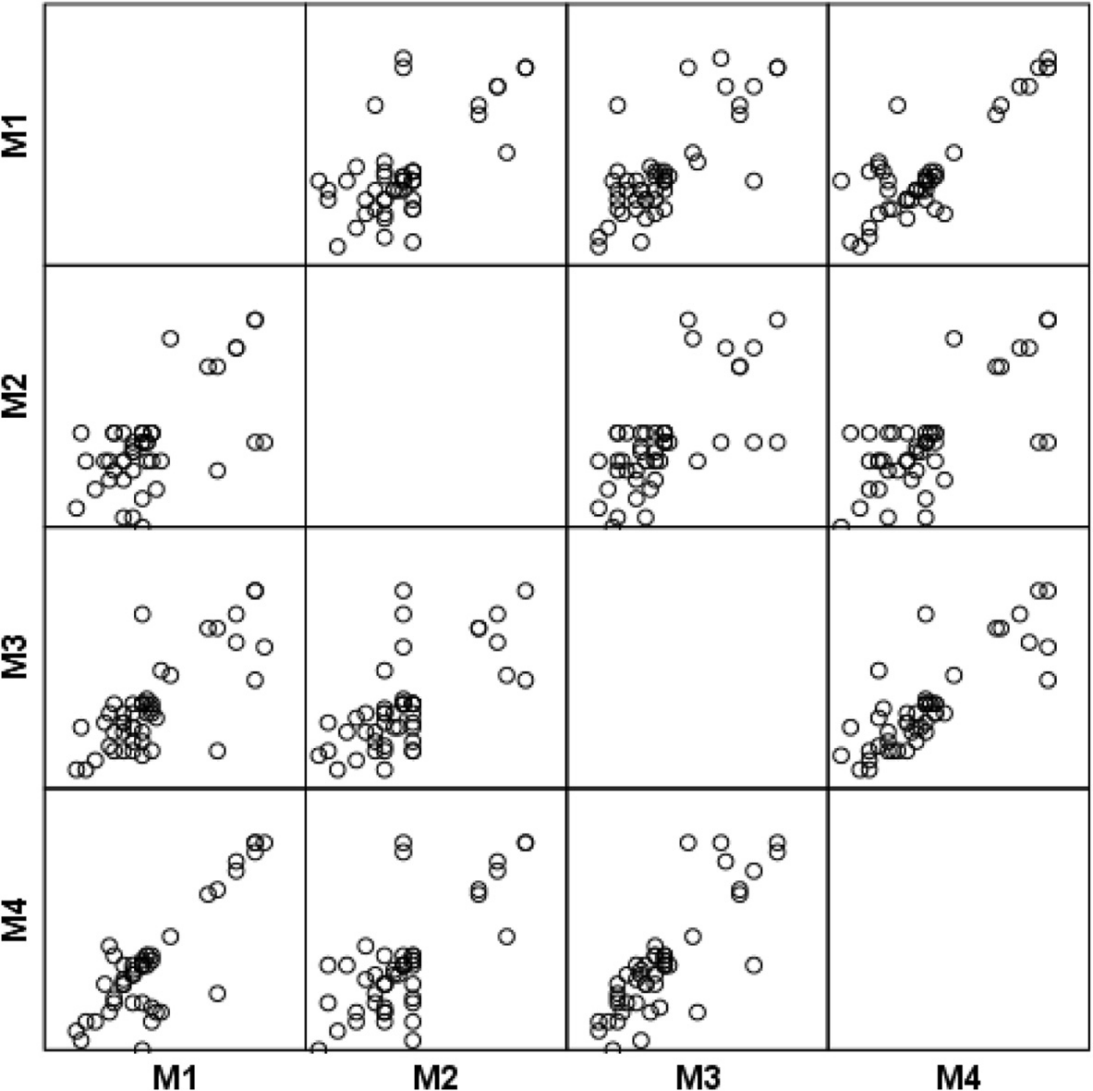
Plots of interclass correlation coefficient between the four different observers (M1 through M4). The mean value was 0.67

### Data analysis

The inter- and intrarater reliability for remote visual assessment of wound reepithelialization was calculated using inter- and intraclass correlation coefficients, respectively. Correlation coefficients with 95 % confidence intervals (CI) were derived from an analysis of variance model regarding the rater effect as random [9]. Remote assessment as well as clinical assessment of reepithelialization were examined for each rater by estimating their correlation and a plot of the difference between remote and clinical values against the clinical value. Similarly to a Bland-Altman plot [10], the difference between remote and clinical values was plotted against the clinical value as references. A linear regression model was then used to analyze the dependency of the deviation of the rater’s assessments and the clinical assessment of time to reepithelialization on clinical assessment.

## Results and discussion

### Intrarater reliability

The intrarater correlation coefficient for the two assessments ranged from excellent values (*r* = 0.99; 95 % CI, 0.99, 1) to moderate values (*r* = 0.51; 95 % CI, 0.27, 0.70). The other two raters had *r*-values of 0.71 (95 % CI, 0.53, 0.83) and 0.97 (95 % CI, 0.95, 0.99), respectively. The mean intraclass correlation coefficient was 0.79 (95 % CI, 0.62, 1.00), representing an excellent value [7].

### Interrater reliability

The interclass correlation coefficient was 0.67 (95 % CI, 0.57, 1.00), including values of all four raters, representing a good value [7] (Fig. 2).

### Validity

**Fig. 3:**
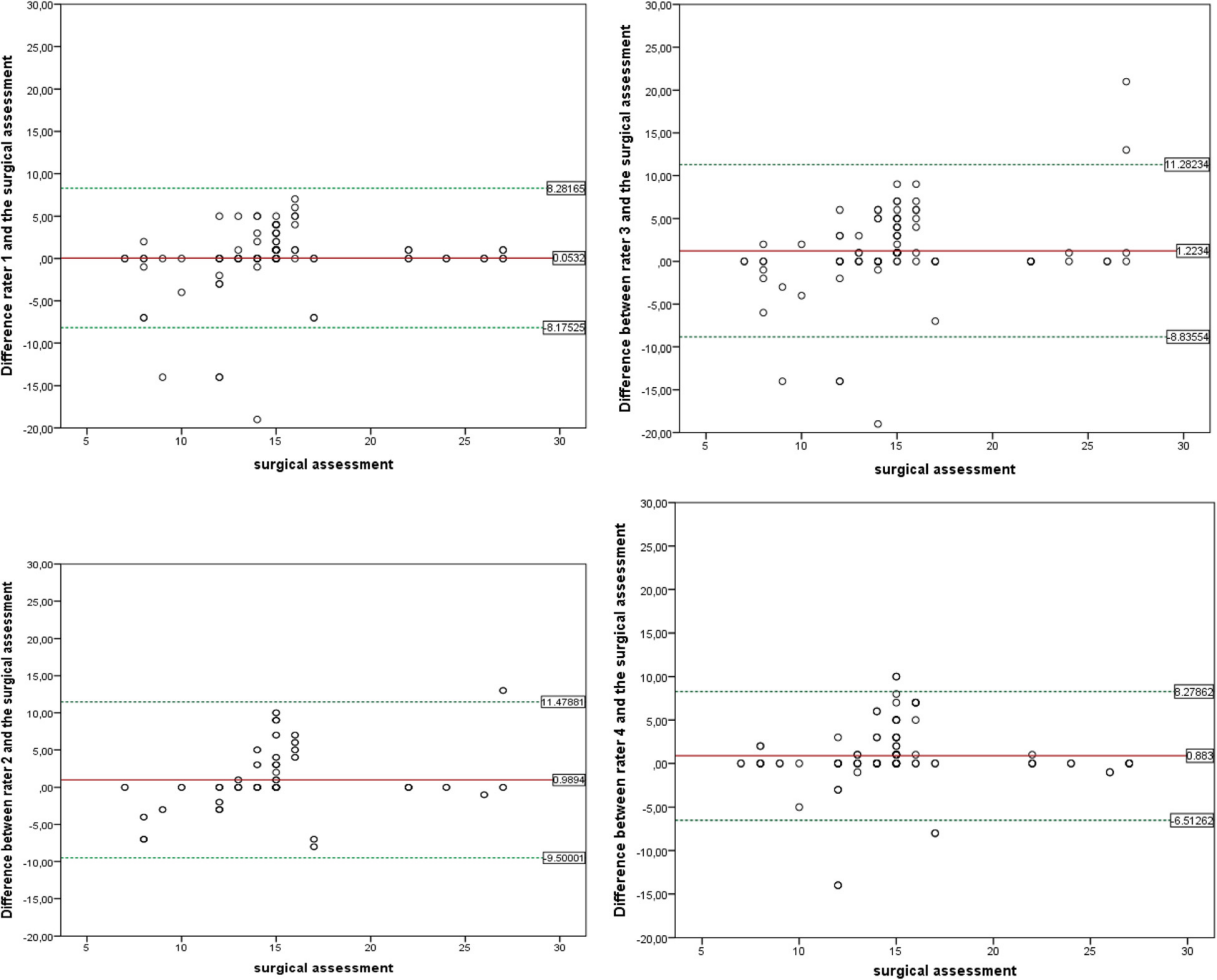
Bland-Altman plots comparing clinical surgical assessment of time to wound closure and remote analysis of photographs by blinded observers

The agreement between remote visual assessment of reepithelialization and direct clinical assessment of reepithelialization at the time of the study was satisfactory. The correlation between rater assessment and clinical assessment was good, with correlation coefficients of 0.67, 0.60, 0.52 and 0.76 for the four raters, respectively. The plots demonstrate an overall difference of approximately 1 additional day by three of four raters (*P* = 0.21, 0.24, 0.31 and 0.90, respectively) (Fig. 3). Moreover, we identified a tendency for an underestimation of the time to epithelialization in cases of wounds that exhibited faster clinical reepithelialization, as well as a tendency toward overestimation in cases of late or protracted reepithelialization. The regression model revealed a dependency of the difference between rater and clinical assessment on the clinical assessment (*P* = 0.008).

The principal goal in the care of patients with cutaneous wounds is achieving reepithelialization. The clinical significance of timely wound closure cannot be overstated, particularly when one considers patients with extensive burn wounds. Increasing or improving the rate of epithelialization may lead to reduced morbidity and mortality [11]. A variety of wound dressings and ointments have been designed to improve the healing of partial thickness wounds.

The efficacy endpoint for most wound-related studies is generally considered to be improved wound healing; however, improved wound healing encompasses a number of different clinical parameters. Improved wound healing can be judged according to the incidence of complete wound closure, accelerated wound closure, facilitation of surgical wound closure and the long-term quality of the resultant healing, be it in form, function or scar formation [12]. For phase III clinical trials, only complete wound closure suffices [12]. A 95 % reepithelialization rate is used and generally accepted as an indicator of complete wound closure in most studies related to clinical wound healing. Newly epithelialized wounds are quite fragile, particularly early on. It is well documented that even minimal sheer can lead to small recurrent wounds. The maturing basement membrane formation and stable anchorage of the newly formed epithelium can take up to 1 month or more, depending upon the depth and extent of the wound, the patient’s age, injury pattern and comorbidities among other factors. This has led to the generally accepted recommendation to follow trial subjects for a period of at least 3 months after closure.

In the field of wound care, clinical research questions are not always amenable to a double-blind clinical study design, widely considered the gold standard for clinical trials. In addition, characteristics of topically applied drugs and devices may render these inaccessible to a double-blind study design. The reasons for the complexity or even impossibility of blinding evaluating clinicians in wound-healing studies are broad. Pain during dressing changes, keeping aseptic conditions during dressing changes or lack of staff are examples for limitations on blinding the evaluating team. Nevertheless, high-quality randomized controlled clinical trial designs can be achieved in wound care using an open trial design. This requires a secure randomization procedure that ensures that the treatment regimen can be assigned only after selection of the patient’s study wound area, and a photography-based, blinded evaluation is generally advocated for the primary endpoint. The present study provides evidence that a study design with photography-based, blinded evaluation by external reviewers can effectively be used to ensure objective analysis. It should be noted that the wound types examined in this study reflect standardized *in vivo* wound types, and future studies might be warranted with respect to other wound type presentations, such as those resulting from trauma, radiation and malignancy. It is generally accepted that the principal goal of most clinical trials is to provide objective, non-biased assessments of study parameters, particularly the primary endpoint. It remains questionable whether subjective clinical assessments of the wound will be accepted as the primary endpoint evaluation in open-label studies. Computer-aided photographic assessment has been shown to be less reliable than expert photographic evaluation, as described by Middelkoop and colleagues [4, 6] and Durani *et al*. [13]. Accordingly, we advocate that photographic evaluation by experts in the field still represents the best available method for studying blinded evaluation of wound-healing progression. In this study, the treatment protocol design delineated wound treatment sites in halves, with identical photographic settings applied to both control and verum groups. In a subsequent phase III trial with use of a mirror-sided wounds camera, camera settings, lens and flash were similarly standardized to the uniform settings at all participating centers.

It should be noted that the number and quality of external reviewers is likely of critical importance. It has been shown that experience of the observer resulted in an increase in reliability [6]. *Experience* was defined by the authors as having experience in wound care, specifically in burn care, greater than 10 years. In our study, we were able to demonstrate that four external reviewers with different clinical backgrounds provide sufficient reliability, as demonstrated by interclass reliability. Evaluating a wound and the degree of epithelialization based only upon a photograph is clearly more challenging than clinical practice, as no information may be available pertaining to several pertinent factors, such as the appearance of the removed wound dressing or odor [1]. In addition, it is important to recognize that a single photograph shows only a single two-dimensional view of the wound. From a clinical perspective, wounds are often regarded from multiple angles in an effort to best assess the wound state. It is interesting to note, in contrast, that Bloemen *et al*. [6] recommended only a single experienced observer for assessment of wounds, although the intraclass correlation coefficient for interrater reliability was 0.66 for the parameter graft take and 0.56 for epithelialization of skin-grafted wounds. However, the authors acknowledged that reliability increases with a second observer. In subsequent phase III, open-label clinical studies on wound healing (EU Clinical Trials Register EudraCT numbers 2012-000777-23 and 2012-003390-26), we utilized three observers and found an excellent intraclass correlation coefficient for interrater reliability (unpublished observations).

Objective measurement of wound epithelialization remains a challenge for both clinicians and researchers. A variety of technical measures have been used to assess epithelialization [1–3]. These include measurement of transepidermal water loss and electrical impedance of the wound. Unfortunately, most of these devices can be used to assess only a single point at a time, as the diameter of aperture is small (<1 cm). Technical noninvasive improvements such as the use of fluorescent dyes might one day be employed clinically to differentiate between an open wound and new epithelium in sufficiently sized wounds, a methodology that would mimic ophthalmologic evaluation of corneal ulceration and healing. The assessment of an entire wound of clinically relevant size is not possible, thus allowing for bias in site section. This inherent bias similarly complicates the generally accepted gold standard objective measurement of reepithelialization—histology [14]. Singer *et al*. [14] reported that agreement between clinical and histological assessments of reepithelialization studied in a porcine partial thickness burn wound model was poor. Single-point analysis of a wound site cannot generally be considered acceptable in clinical trials. In an effort to obtain a less biased assessment of wound healing, multiple biopsies are often advocated. It should be recognized, however, that these additional wound site samples taken at various time points must to some degree disturb overall wound healing. The challenges of whether patients will consent to participate in a study with multiple sequential biopsies further complicates its clinical application. It is important to note, however, that the clinical assessment used for baseline comparison in the present study is of highest practical relevance, as it reflects a direct consequence for treatment decisions (wound dressing renewed or no longer required).

It was interesting to note that remote photographic assessment underestimated wound reepithelialization as compared with clinical observation (positive difference). This fact will not influence the value of clinical trials, as remote assessment of wound closure correlates well with direct clinical assessment by the clinician. Bloemen *et al*. reported findings similar to ours regarding donor site wounds. They found a strong correlation between clinical assessment and digital image analysis of reepithelialization in split-thickness skin-grafted wounds [6]. As mentioned above, photographs are at present only two-dimensional and are established from a single perspective, whereas in clinical practice more views from different angles provide more information.

## Conclusions

Remote photographic analysis of cutaneous wounds is a feasible method of evaluating time to complete (>95 %) wound closure in open-label clinical studies. Remote analysis of photographic images is a reliable method of wound closure measurement and correlated satisfactorily with clinical judgment, bolstering its potential value in the current era of evolving application and dependency in the field of telemedicine.

## Abbreviation

CI: Confidence interval
